# Supplementary material for: The Preschool Eating, Lifestyle, and Sleeping Attitudes Scale (PRELSA Scale): Construction and Pilot Testing of a Tool to Measure Factors Associated with Childhood Obesity
Source: Healthcare (Basel). 2023 May 9;11(10):1365. doi: 10.3390/healthcare11101365 (PMC10218310; doi:10.3390/healthcare11101365)
Supplement: Supplementary file 1 [file healthcare-11-01365-s001.zip › healthcare-2317559-supplementary.pdf]

Table S1: Demographic Characteristics in Families Pilot Test

Table S2: Demographic Characteristics in Experts Pilot Test

Table S3: Qualitative Information of Experts during Pilot Test

Table S1: Demographic Characteristics in Families Pilot Test

Variable	Categories	n	%
Sex	Women	23	88.5
	Men	3	11.5
Marital Status	Married	22	84.6
	Divorced	1	3.8
	Single	3	11.5
Education Level	Secondary	3	11.5
	Bachelor	3	11.5
	Professional Training	5	19.2
	University Degree	9	34.6
	Master	3	11.5
	PhD	3	11.5
Job Situation	Full Time Job	16	61.5
	Partial Time Job	8	30.8
	Unemployed	2	7.7
Income	1000€-2000€	12	46.2
	2000€-4000€	9	34.6
	More than 4000 €	5	19.2
Home Place	Urban	15	57.7
	Countryside	11	42.3
Outdoor spaces	Yes	18	69.2
	No	8	30.8
Feeding Responsible	Yes	12	46.2
	Equal shares with another person	12	46.2
	No	2	7.7
Care Responsible	Yes	14	53.8
	Equal shares with another person	12	46.2
	Mean	SD	IC
Age	36.5	4.4	(27.8, 45.1)
Size of the Home (in m^2)	133.3	77.7	(-18.9, 285.5)
Family Members	3.6	1.7	(0.3, 6.9)
Children Age	3.1	1.0	(1.2, 5.1)

Table S2: Demographic Characteristics in Experts Pilot Test

Variable	Categories	n	%
Sex	Women	18	72.00
	Men	7	28.00
Job	Pediatrician	9	36.00
	Physician (Not pediatrician)	5	20.00
	Nurse	5	20.00
	Researcher	6	24.00
	Mean	SD	IC
Age	49.4	10.3	(29.2, 69.6)
Experience (years)	16.9	6.7	(3.8, 30.1)

Table S3: Qualitative Information of Experts during Pilot Test

General Opinion	About Items	Aditional Information	Response
The questionnaire is quite comprehensive. It does get a bit extensive, but as stated above, it is a tool to create a more accessible scale.	I do not consider it.		*Thank you very much for your answers and cooperation*
It is extensive, but necessary as a basis for developing a reduced scale.	I consider that it is balanced in the different aspects it analyzes. Since it is a scale that evaluates so many aspects of preschool feeding and lifestyles, the number of items it contains is necessary. Nor would a larger number give better information.		*Thank you very much for your answers and cooperation*
It should make the possible answers to the questions very clear, otherwise it can create confusion.	It would be necessary to incorporate questions addressed to the adults in the family about their degree of commitment to being an example for the children in each of the items.		*Thank you very much for your answers and cooperation. With respect to your suggestion, there are several items already on the scale associated with parental support for these attitudes*
The questionnaire is well elaborated. The questions are well formulated, but it is very long.	Yes, some redundant items should be eliminated so that they can be answered in less time.	Some blocks of questions are preceded by a title that can induce the sense of the answer.	*Thank you very much for your answers and cooperation. In this first version, the length is normal due to the objectives set. Regarding your suggestion, we have eliminated 2 items for being redundant and the title of each block of questions.*
I think it can be very useful in identifying a child population susceptible to poor eating and health habits.			*Thank you very much for your answers and cooperation*
It seems to me to be an accurate, reliable instrument, adapted to the environment in which we work, which will undoubtedly be very useful.	I think it is a very complete document		*Thank you very much for your answers and cooperation*
The wording of some questions that are not well formulated should be modified.		From the first section both in question 25 and 26 do not collect always, rarely, sometimes if not quantify.<br>In section 2 in restrictions specify in the question I consider it appropriate to keep some foods out of reach of my child. Specify which.<br>In the part of physical activity in AAF1A remove studies because it is already repeated in EAF1B.<br>Question EAF4 is not understood.<br>In question HS2 specify what type of reward.<br>In the third section of questions 1 to 11, do not use rarely, frequently... but quantify.	*Thank you very much for your answers and cooperation. Your suggestions have been taken into account to make several changes, mainly regarding the standardization of the way of answering the questions.*

		In the next 9 questions also do not use rarely... yes no yes or no or quantify.<br>In question 2 specify whether it is expressed in hours or minutes.	
		There are certain items that I think need to be more structured. For example, those referring to the generic information of whether they suffer from any pathology (without specifying which child suffers from it... since there could be more than one).	*Thank you very much for your answers and cooperation. In the online version of the survey this has now been fixed.*
It seems to me to be an appropriate questionnaire although it is very extensive	Reduce the number of questions in each item to facilitate the completion of the questionnaire, covering all items with fewer questions.	A very complete survey. Congratulations	*Thank you very much for your answers and cooperation. In this first version the length is normal due to the objectives set out.*
I think it is very good, but some questions are posed in a way that is not very easy to understand.			*Thank you very much for your answers and cooperation. We have tried to modify the wording of the items to improve the understanding.*
I think it is very complete and interesting to analyze attitudes and behaviors.	22a) Indicate the other pathology associated with the children in the previous question. This question is not well understood: if you refer to another pathology, remove the article la and put: another pathology not included in the previous question.		*Thank you very much for your answers and cooperation. This has been fixed in the online version of the survey.*
I think it is excellent, perhaps too exhaustive	Very complete		*Thank you very much for your answers and cooperation.*
It is very complete; it reviews all the items of interest for the object of study.	I don't know which one could be eliminated, they all seem very relevant to me. Although it may be long for some unaccustomed parents.	It may be long for some parents. On the other hand, answering the questionnaire can reveal some parenting mistakes.	*Thank you very much for your answers and cooperation. In this first version the length is normal due to the objectives set out*
It seems to me to be an appropriate questionnaire although it is very extensive,<br>I think it is very good, but some questions are posed in a way that is not very easy to understand.<br>I think it is very complete and interesting to analyze attitudes and behaviors.<br>I think it is excellent, perhaps too exhaustive.<br>It is very complete, it reviews all the items of interest for the object of study.<br>The questionnaire covers very satisfactorily the main factors associated with childhood obesity. In addition, as a tool for research use it is very complete and useful. However, the current structure is not friendly to be self-completed by parents, who will	Yes, the survey in the eyes of the general population is quite long and repetitive. Also, in some sections it can be ambiguous for parents to answer. For example, when asked about screen use while the child eats, the question asks. "I consider it appropriate for my child to eat while being entertained by a screen". It may be the case that parents do not consider it appropriate, but still allow their children to eat with them. In this case, for example, parents may have doubts about what they should answer. So in these cases the information collected loses quality and usefulness because the belief is in line with reality.	For a self-completed questionnaire the structure should be more friendly and easy to fill in. People lose interest quickly, so it is ideal to always state the possible answers under each question. This way, they don't have to go back to the top to see the possible answers and it also allows them to answer quickly by marking with an X or similar.	*Thank you very much for your answers and cooperation. The information provided will be taken into consideration and some items will be modified. Two questions have been added to the parents to know their BMI and their specific lifestyle habits. In addition, the questionnaire as it is organized in the online version makes the structure more user friendly as stated in the comment.*

be of different socio-educational levels. In addition, the questionnaire in its current version obviates very important details in the habits of very young children, which are the habits of the parents or main caregivers. As a researcher, I have frequently analyzed factors associated with the consumption of ultra-processed foods, consumption of sugar-containing beverages, level of physical activity and obesity, among others, in children aged 4-5, and the factors most strongly associated with these habits in the preschool population are always the habits of the parents (dietary, physical activity, socioeconomic level and weight status). There is quite a lot of literature published on this subject. If some questions on parental/caregiver weight status and certain dietary/physical activity/sleep habits are not included, I believe that the survey will be incomplete.			
For some parents it may be too long and it has quite a few questions that are very similar, but with some difference that I don't know if they will know how to answer adequately.	I don't remember if they asked about the lunch at recess ...they usually opt for something quick and that the children like.	I think it's a very good job. There is a lot of childhood obesity	*Thank you very much for your answers and cooperation. Some items have been modified to avoid possible redundancies.*
I consider that its objective is more than covered with the questions it asks. Perhaps the explanation given to the parents could be somewhat improved.	I consider that it more than covers the dimensions, although as it is a longer first version, some very similar questions are sometimes repeated.		*Thank you very much for your answers and cooperation. In this first version the length is normal due to the objectives set out*
I think it is extensive but adequate to obtain the expected data, the sections appear to me to be adequate.	I think it would be interesting to include in the questionnaire if the patient has received treatment with inhaled corticosteroids for more than 6 months a year, as asthma and bronchitis in the first two years of life is the most prevalent pathology at this age, the prolonged use of corticosteroids is a fact to be taken into account.<br>Another interesting question would be if the children eat in the dining room if they complement the dinners with the indications of the nutritionists who elaborate them? If they do not eat in the dining room, do they have a weekly meal plan? This facilitates the elaboration of healthier and healthier meals, avoiding the improvisation that is always precooked meals or orders of meals at home. I don't know how to ask but I advise my patients to	Offer web links to be able to read and be informed in child nutrition, to be able to direct to reliable sources more and more parents seek information on social networks and end up watching or following youtubers with no training on the subject.	*Thank you very much for your answers and cooperation. In this first version the length is normal due to the objectives set. We will take into account several of your recommendations. We have incorporated questions about where children eat at lunchtime and added links to official information on childhood obesity at the end of the online survey.*

	elaborate a weekly menu to better distribute the caloric intake.<br>It can be interesting in the intervention to offer easy workshops or tools to elaborate it. On the other hand, this facilitates the shopping list and avoids repeating foods that are always the ones that children prefer: pasta, pizza and hamburgers for example.		
Very complete, it touches on all the necessary variables. As a pilot to later make a reduced version, analyzing which items are redundant and what number of items would be essential, I think it is good. It can be a bit tedious to fill out, especially for low educational levels.	There are items that can generate confusion, those that agglutinate several possibilities of response. For example, if they participate in breakfast, lunch, etc., it may be that they never participate in lunch but always do so at dinner, it will then be difficult for them to place themselves on the scale and may induce intermediate answers.		*Thank you very much for your answers and cooperation. In this first version the length is normal due to the objectives set. Regarding the way to measure meal participation, we have modified the way to ask it in the final version.*
The questionnaire is too long. There are too many items to complete.	Reduce Items	It would be convenient to try to match the type of response of the items, not to leave open answers but always try to generate multiple choice answers.	*Thank you very much for your answers and cooperation. In this first version, the length is normal due to the objectives set. The answers of the items of the scale have been equalized in the new version according to your suggestion.*
The data collected will undoubtedly offer a broad perspective that will allow a risk profile and intervention proposals to be made.	If the aim is to create a shorter and therefore more practical tool, this broader format may be the way to visualize how it should be approached and which items are essential beyond the cataloging of data.	For the application of the definitive tool, it should be considered that the time of a scheduled consultation in primary care is 15-20 minutes and that of clinical demand is 8-10 minutes in pediatrics.	*Thank you very much for your answers and cooperation. In this first version the extension is normal due to the objectives set.*
Good questionnaire. It does not include an assessment of the consumption of whole grains in the diet, which is important to avoid obesity.	Incorporate assessment of consumption of whole-grain bread, pasta, rice and other whole-grain cereals.		*Thank you very much for your answers and cooperation. The evaluation of the consumption of these foods, although interesting, is beyond the scope of this questionnaire.*
